# Efficacy, safety, and quality of life profile of Genotype-3 Chronic Hepatitis-C Pakistani patients receiving ledipasvir plus sofosbuvir treatment

**DOI:** 10.12669/pjms.40.7.7869

**Published:** 2024-08

**Authors:** Zahid Yaseen Hashmi, Sandeed Hashmi, Ali Raza

**Affiliations:** 1 Dr. Zahid Yaseen Hashmi, FCPS Medicine. Chairman Liver Foundation Trust, Faisalabad, Pakistan; 2 Dr. Sandeed Hashmi, MBBS. Liver Centre Faisalabad, Pakistan; 3 Dr. Ali Raza, MBBS. Liver Centre Faisalabad, Pakistan

**Keywords:** Genotype-3, HCV, Health-Related Quality Of Life, Sofosbuvir, Ledipasvir, Ribavirin

## Abstract

**Objective:**

This study aimed to assess the overall treatment response of Genotype-3 Chronic HCV Pakistani Patients with or without cirrhosis to Ledipasvir plus Sofosbuvir combination.

**Method:**

In this observational study, HCV Genotype-3 patients were enrolled from Liver Center, DHQ Hospital, Faisalabad and divided into two groups, i.e., non-cirrhotic and compensated cirrhotic patients. The study spanned for a period of 24 months (November 2019 - November 2021) from the first enrollment to the last follow up. Non-cirrhotic patients received Ledipasvir/Sofosbuvir (LDV/SOF) 90/400mg for 12 weeks and cirrhotic patients received LDV/SOF with Ribavirin (RBV) for 12 weeks and without RBV for 24 weeks. The treatment efficacy in terms of sustained virological response (SVR12) was monitored 12 weeks post-treatment. The safety profile, and health-related quality of life (HRQoL) were monitored from baseline to follow-up visits.

**Results:**

Two hundred and ninety out of 309 (93.85%) non-cirrhotic and 31 out of 33 (93.94%) compensated cirrhotic patients achieved SVR-12. The safety profile of the non-cirrhotic and compensated cirrhotic patients was comparable throughout the study duration. Fatigue was the most commonly reported adverse event (AE) in non-cirrhotic and compensated cirrhotic patients, followed by headache, nausea, and fever. The HRQoL improved from baseline to follow-up visits among patients of both groups.

**Conclusion:**

It is concluded that LDV and SOF combination regimen is safe and effective for treating Genotype-3 HCV patients without cirrhosis/compensated cirrhosis, and also improves the patient’s HRQoL.

## INTRODUCTION

According to World Health Organization (WHO), HCV has affected 71 million people globally.^1^ Community-based prevalence surveys of HCV have not been done in the recent past. The only data available is from the Pakistan Medical Research Center (PMRC) from July 2007 to May 2008, showing 7.8 million Pakistani population at that time.^2^ Pakistan is believed to have the second highest number of HCV patients.^3^ A global epidemiology survey has reported that HCV prevalence in Pakistan is 5.8% ranging from 1.4% to 8.7%, and 79% of them are Genotype-3 infection.^4^ The progression of fibrosis, prevalence of severe steatosis, and incidence of hepatocellular carcinoma is higher among the individuals with HCV Genotype-3 as compared to other genotypes.^5^

In the current direct-acting antiviral (DAA) therapy era, HCV Genotype-3 infection lagged behind other genotypes, especially among patients with cirrhosis or prior HCV treatment failure. The combination of antiviral agents with higher SVR, shorter duration of treatment and separate targets for possible interferon-free regimens are under evaluation.^6^ LDV and SOF inhibits HCV NS5A protein and NS5B RNA-dependent RNA polymerase, respectively, essential for viral replication, assembly, and secretion. SOF is a nucleotide prodrug; it is metabolized to the active triphosphate nucleoside analog by sequential hydrolysis, phosphoramidite cleavage, and phosphorylation in the liver. SOF, incorporated into HCV RNA by NS5B polymerase, acts as a chain terminator.^7^

The efficacy and safety of the LDV/SOF combination have been demonstrated in several well-designed clinical trials, and it is found to be well-tolerated. The reported AEs include headache, insomnia, fatigue, nausea, dizziness, pruritis, upper respiratory tract infections, rash, etc.^8^-^11^ In addition to the disease condition, the patient’s Quality of Life (QoL) is also influenced by the treatment modalities. In the past, treatment with interferon-based therapy was associated with varying degrees of AEs such as fatigue, myalgia, flu-like symptoms, blood dyscrasia, alterations in mood, mild to severe depression, and even frank psychosis, which would negatively affect the HRQoL of the patients in terms of changing their vitality, social interaction and ability to function at work.

However, the LDV/SOF-associated AEs are few compared to the interferon treatment regime.^12^-^15^ The objective of the present study was to assess the overall response of Genotype-3 Chronic Hepatitis C Pakistani Patients with or without cirrhosis to LDV/SOF ± RBV treatment in terms of safety, efficacy, and quality of life of the patients from baseline to the follow-up visit.

## METHODS

This observational single-center study was conducted at Liver Center, DHQ Hospital, Faisalabad for a period of 24 months (November 2019 - November 2021) from the first enrollment to the last follow up to assess the efficacy, safety, and quality of life of Genotype-3 Chronic Hepatitis-C patients with or without cirrhosis receiving LDV/SOF ± RBV treatment.

### Ethical Approval:

The Pakistan Medical Association approved the study protocol (Ref No. AM/942/PMC/15) and again the protocol was reviewed by the Institutions Ethics Committee which approved it. Ref. IC/ERB/03/2019 Dated July 11, 2019.

The sample size was calculated using WHO software for sample size determination in health studies, considering 95% confidence level, 5% point absolute precision, and SVR12 rates in patients without cirrhosis p1=50% and compensated cirrhosis p2=50%, and intermediate value of 0.26, a sample size of 400 was caluclated.^10^ Non-probability sampling technique was used for the patient selection and Patients’ written authorization was obtained at the time of enrollment, and data secrecy was assured and maintained as per ICH-GCP guidelines. All patients with chronic HCV Genotype-3, without cirrhosis, with compensated cirrhosis (Child-Pugh Class A), treatment naïve (TN) or treatment experience (TE) (naïve to ledipasvir plus Sofosbuvir combination treatment), and aged ≥ 18 years were included in this study. Patients with HIV or HBV co-infection, those diagnosed with decompensated liver disease (Child-Pugh Class B & C), liver transplant, thalassemia, uncontrolled diabetes, uncontrolled hypertension, CVD, patient with eGFR < 30 mL/min/1.73 m^2^, receiving amiodarone, with known hypersensitivity to Ledipasvir, Sofosbuvir, and Ribavirin, pregnant and lactating women were excluded from the study.

The enrolled Genotype-3 HCV patients were divided into two groups; Group-1 included non-cirrhotic patients and Group-2 compensated cirrhotic patients. Non-cirrhotic patients received Ledipasvir/Sofosbuvir (LDV/SOF) 90/400 mg for 12 weeks and cirrhotic patients received LDV/SOF with RBV (weight-based ribavirin < 75 kg = 1,000 mg and ≥ 75 kg = 1,200 mg, administered orally in two divided doses with food) for 12 weeks and without RBV for 24 weeks. The enrollment was based on the diagnosis of Chronic Hepatitis-C (CHC) and the prescription of study medication.

In addition to the baseline characteristics (clinical history, demographics, laboratory investigations, signs, and symptoms), the sustained virological response was monitored 12 weeks post-treatment. The safety analyses and health-related quality of life (assessed via SF-36 V2) were recorded throughout the follow-up.

### Statistical Analysis;

It was performed on SPSS version 22.0; descriptive statistics were used to present the baseline data where qualitative data were expressed as frequencies with percentages and quantitative data as mean with standard deviation or median with IQR. The change in the laboratory examination and safety profile from baseline to follow-up visits was assessed using the Chi-square/fisher exact test, paired sample t-test, and one-way analysis of variance (ANOVA) as appropriate. The mean difference in Quality of Life from baseline to follow-up visits was analyzed using repeated-measures ANOVA. A p-value < 0.05 was considered significant.

## RESULTS

**Fig.1:**
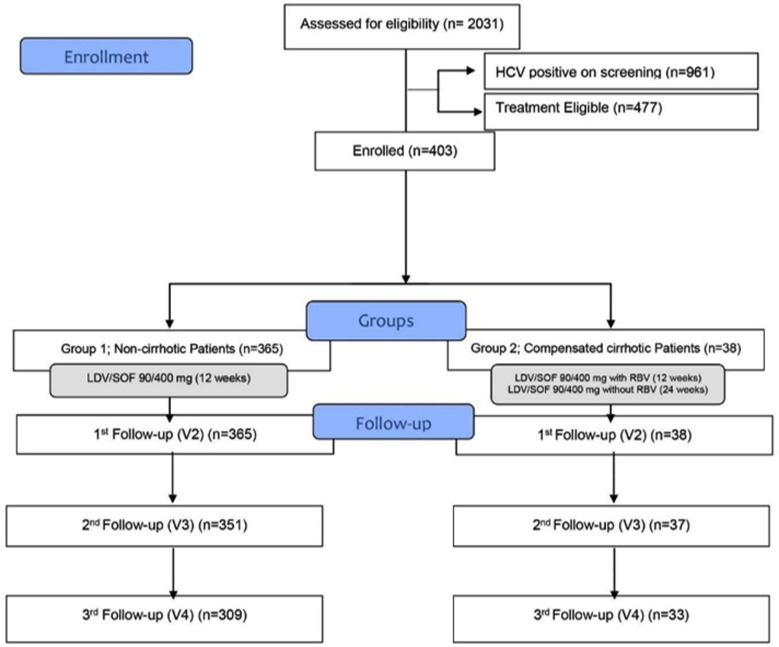
Study Flow.

The mean treatment duration was 13.2 ± 5.2 weeks. Of the 477 eligible patients, 403 were enrolled and received LDV/SOF (90/400 mg) treatment. There were 365 non-cirrhotic and 38 compensated cirrhotic patients. In total, 309 non-cirrhotic and 33 compensated cirrhotic patients completed all three follow-up visits (Fig.1). It was found that 93.85% (290 out of 309) of the non-cirrhotic and 93.94% (31 out of 33) compensated cirrhotic achieved SVR12. There was no significant difference in the safety profile of the non-cirrhotic and compensated cirrhotic patients, except the observed events related to nausea in the two groups (p=0.019). The observed adverse events were mild; dose changes, treatment correction, and discontinuation were carried out as per the physician’s recommendation (Table-II). There was a significant improvement in almost all the quality of life sub-scales from baseline to follow-up visits among non-cirrhotic and compensated cirrhotic patients, except a few. No significant difference was observed in pain from baseline and visit 2 (1^st^ Follow-up) among non-cirrhotic patients. While compensated cirrhotic patients displayed no change in physical functioning, energy/fatigue, emotional well-being, and general health from baseline and visit 2 (1st Follow-up) (Table-III).

**Table-I:** Baseline demographics and clinical characteristics.

Variables			Group 1		Group 2		p-value

			(n=365)		(n=38)		

			Median	IQR	Median	IQR	
Demographic & Clinical Characteristics	Age; years		43.0	35.0-50.0	40.0	34.7-50.0	0.442
	Height; cm		156.0	150.0-164.0	155.0	149.5-160.5	0.265
	HbA1c (%)		9.2	55.0-74.0	9.2	7.0-11.3	0.433
	Gender; n(%)	Male	139	38.1	10	26.3	0.153
		Female	226	61.9	28	73.7
	Weight; kg		65.0	55.0-74.0	64.5	51.5-74.5	0.982
	BMI; kg/m^2^		25.0	21.6-28.0	24.0	21.0-29.8	0.779
	SBP; mmHg		110.0	110.0-120.0	110.0	100.0-120.0	0.861
	DBP; mmHg		70.0	70.0-80.0	70.0	70.0-80.0	0.588
Laboratory Examination	Hb; g/dL		13.2	12.0-14.3	13.1	12.0-13.9	0.129
	Total RBC; %		4.8	4.3-5.2	4.6	4.2-5.1	0.228
	MCV; Fl		81.8	78.1-86.5	85.5	79.7-90.2	0.069
	MCG; pg		28.3	25.8-29.5	28.7	27.3-31.6	0.69
	MCHC; g/dl		33.9	32.0-34.4	33.2	32.2-34.6	0.484
	Platelet count; x 10^9^/l		252	207-304	153	133-271	0.017*
	WBC count; x 10^9^/l		7.9	6.6-8.9	7.0	5.7-8.4	0.251
	Neutrophils; %		51.5	49.0-61.0	59.5	56.8-65.3	0.001*
	Lymphocytes; %		37.5	32.0-43.0	35.0	30.3-39.8	0.08
	Monocytes; %		3.5	2.5-5.0	2.5	2.0-3.5	0.005*
	Eosinophils; %		3.5	2.5-5.0	3.0	2.0-4.0	0.266
	Serum creatinine; mg/dL		0.9	0.8-1.0	0.9	0.8-1.0	0.154
	ALT; U/L		54.0	38.0-88.0	68.0	40.0-110.8	0.793
	AST; U/L		47.0	36.0-71.0	65.0	38.0-105.0	0.045*
	ALP; U/L		220.5	176.2-272.7	279.5	230.8-299.5	0.011*
	Albumin; g/dL		4.3	4.0-4.4	4.1	3.8-4.2	0.013*
	Bilirubin; mg/dL		0.9	0.7-1.1	0.9	0.6-1.2	0.848
	INR		1.0	-	1.0	-	0.688

Group-1: Non-cirrhotic patients; Group-2: Compensated cirrhosis patients.

* p<0.05 is considered significant.

**Table-II:** Assessment of Safety of LDV/SOF ± RBV from baseline to the follow-up visit.

Variable	Group 1	Group 2	p-value
Fever	124(14.62)	15(13.04)	0.644
Headache	153(18.04)	22(19.13)	0.142
Anxiety	34(4.01)	9(7.83)	0.152
Insomnia	67(7.90)	9(7.83)	0.358
Fatigue	339(39.98)	40(34.78)	0.651
Nausea	114(13.44)	17(14.78)	0.019*
Diarrhea	17(2.00)	3(2.61)	0.260

Group-1: Non-cirrhotic patients; Group 2: Compensated cirrhosis patients. Values are given as frequency. Normal range for Platelet-150 to 400 × 10^9^/L; ALT - 7 to 55 units per liter.

* p<0.05 is considered significant. **All the above-mentioned AEs were non-serious-mild or transient discomfort, and did not require intervention or treatment.

**Table-III:** Quality of life from baseline to the follow-up visit.

Variables	1st Follow-up Visit	Last follow-up Visit	Mean Difference	95% CI		p-value

				Lower	Upper	
***Non-cirrhotic patients***						
Physical functioning	65.14±12.24	50.30±22.43	14.83	12.30	17.37	0.000*
Role limitations due to physical health	43.39±22.30	25.07±23.00	18.32	15.50	21.13	0.000*
Role limitations due to emotional problems	40.57±23.91	22.56±22.23	18.01	15.23	20.79	0.000*
Social functioning	41.40±17.65	36.92±20.59	4.48	2.07	6.90	0.000*
Pain	39.20±27.61	52.17±32.32	-12.97	-16.96	-8.98	0.000*
Energy/fatigue	51.96±18.32	40.72±22.52	11.23	8.53	13.94	0.000*
Emotional well-being	48.35±13.50	35.25±17.77	13.10	11.01	15.19	0.000*
General Health	47.77±12.00	44.89±20.06	2.88	0.60	5.16	0.013*
***Compensated cirrhotic patients***						
Physical functioning	63.70±10.12	48.36±26.04	15.34	6.463	24.21	0.001*
Role limitations due to physical health	37.34±20.57	17.60±21.39	19.73	12.532	26.94	0.000*
Role limitations due to emotional problems	35.75±24.26	13.82±18.71	21.93	14.629	29.23	0.000*
Social functioning	42.76±20.26	36.51±22.01	6.25	-3.338	15.83	0.195
Pain	48.43±27.61	50.66±35.10	-2.22	-4.352	9.90	0.712
Energy/fatigue	44.41±16.61	38.49±24.16	5.92	-1.912	13.75	0.134
Emotional well-being	44.21±12.11	31.45±17.58	12.76	6.750	18.77	0.000*
General Health	48.68±12.39	42.80±23.87	5.88	-1.665	13.44	0.123

* Significant difference at p<0.05.

**Fig.2:**
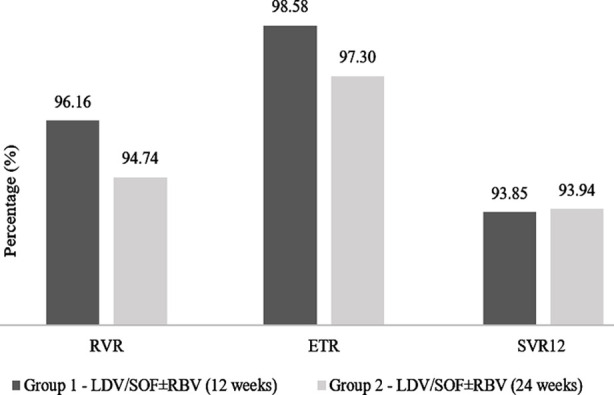
Virological response at follow-up visits. Group 1: Non-cirrhotic Group; Group 2: Compensated cirrhosis group. Rapid Virological Response (RVR); End Treatment Response (ETR), Sustained Virological Response (SVR).

## DISCUSSION

In addition to the safety and efficacy, the current study also evaluated the overall quality of life of the HCV Genotype-3 patients treated with Sofosbuvir/Ledipasvir combination with or without Ribavirin. It was observed that 93.85% of the non-cirrhotic patients who received SOF/LDV ± RBV (12 weeks) and 93.94% compensated cirrhotic patients given SOF/LDV ± RBV (24 weeks) achieved SVR12. Contrastingly existing literature suggest that the presence of cirrhosis limits SVR12 rates.^16^ One of the reasons for this variation might be the difference in the sample sizes of the two groups.

The efficacy of DAAs combinations is comparatively low for Genotype-3 HCV patients than the other genotypes, as demonstrated by the existing literature. Del Rio-Valencia et al. found that the most effective treatment in HCV genotype patients with cirrhosis was SOF + DCV followed by LDV/SOF + RBV for 12 weeks; it was reported that 90% and 80% achieved SVR12, respectively, and it was achieved in all 100% non-cirrhosis patients.^17^ Similarly, Ramos et al. also reported that SVR12 was achieved in 93.3% of HCV Genotype-3 patients.^18^ This difference may be due to the fact that both Del Rio Valencia and Rmaos et al. treated the majority of patients with SOF + DCV, while the treatment regimen used in the present study involved only SOF/LDV ± RBV.

There was no significant difference in the safety profile of the non-cirrhotic and compensated cirrhotic patients. The observed adverse events were mild; fatigue was the most commonly reported adverse effect observed in non-cirrhotic and compensated cirrhotic patients, followed by headache, nausea, and fever. Dose changes, treatment correction, and discontinuation were carried out as per the physician’s recommendation. AbouBakr et al. reported that most patients had headaches, drowsiness, and fatigue, while a few complained of nausea, vomiting, chest pain, and abdominal pain.^19^ Furthermore, none of their patients required drug discontinuation. A systematic review also supported that headache followed by fatigue is the most common side effect in children and adolescents treated with SOF/LDV.^20^

In addition to clinical efficacy (SVR12) and safety, the present study also assessed the impact of these regimens on patients’ HRQoL. There was a significant improvement in the quality of life of non-cirrhotic and compensated cirrhotic patients, as observed through SF-36 V2 subscale scoring from baseline to ETR. However, no significant difference was observed in pain scores from baseline and visit two in the four weeks among non-cirrhotic patients. While compensated cirrhotic patients displayed no change in physical functioning, energy/fatigue, emotional well-being, and general health scores from baseline and visit two. There are both supporting^21^ and contrasting studies in relation to improved quality of life at SVR12-24 with DAAs treatments.^22^,^23^

In a study assessing the quality of life via SF-8, it was found that general health, role emotional, and mental health scores significantly improved from baseline to end of treatment. Whereas the variation from baseline to SVR24 slightly differed, i.e., general and mental health scores were significantly improved while the physical and mental component score remained unchanged. Similarly, Fagundes et al. reported significant improvement in all domains except pain and emotional limitations baseline to ETR.^24^

The study contributes to medical decision-making by providing a detailed view of both clinical and quality of life outcomes. The emphasis on HRQoL outcomes underscores the importance of patient-centric care, acknowledging that successful therapy goes beyond achieving virological cure to embrace the total well-being of individuals. For individuals with Genotype-3 HCV, the combination of Sofosbuvir and Ledipasvir may prove to be a practical and economical choice in areas where obtaining and affording indicated drugs presents difficulties.

### Limitations:

This open-label interventional study included a small sample size; large-scale, randomized trials are required to establish the reliability of these outcomes. Further, due to unequal group sizes, it was not possible to draw inferences in relation to the group-wise comparisons.

## CONCLUSION

In summary, treating Genotype-3 HCV infection patients with the combination of SOF and LDV is safe and effective. It is an alternative therapy to Genotype-3 patients. It not only benefited in terms of clinical outcomes but also improved patients’ HRQoL. Although small yet significant number of G3 HCV related patients fail to respond to the recommended therapies. Besides, most patients typically cannot access or afford the recommended medications. We believe sof/ledipasvir combination may provide an economical alternative that needs to be further investigated.

### Authors Contribution:

**ZYH:** Substantial contributions to conception and design, or acquisition of data, or analysis and interpretation of data.

**ZYH**, **SH** and **AR:** Drafting the article or revising it critically for important intellectual content.

**ZYH:** Final approval of the version to be published.

**ZYH**, **SH** and **AR:** Agreement to be accountable for all aspects of the work in ensuring that questions related to the accuracy or integrity of any part of the work are appropriately investigated and resolved.

## References

[1] Hepatitis C (2017). World Health Organization.

[2] Qureshi H, Bile KM, Jooma R, Alam SE, Afridi HUR (2010). Prevalence of Hepatitis-B and C viral infections in Pakistan:findings of a national survey appealing for effective prevention and control measures. East Mediterr Health J.

[3] Moin A, Fatima H, Qadir TF (2018). Tackling hepatitis C-Pakistan's road to success. Lancet.

[4] Gower E, Estes C, Blach S, Razavi-Shearer K, Razavi H (2014). Global epidemiology and genotype distribution of the hepatitis C virus infection. J Hepatol.

[5] Kanwal F, Kramer JR, Ilyas J, Duan Z, El-Serag HB (2014). HCV Genotype-3 is associated with an increased risk of cirrhosis and hepatocellular cancer in a national sample of U. S. Veterans with HCV. Hepatology.

[6] Kowdley KV, Lawitz E, Crespo I, Hassanein T, Davis MN, DeMicco M (2013). Sofosbuvir with pegylated interferon alfa-2a and ribavirin for treatment-naive patients with hepatitis C genotype-1 infection (ATOMIC):An open-label, randomised, multicentre phase 2 trial. Lancet.

[7] Gilead Sciences Inc (2019). Harvoni^®^ (ledipasvir and sofosbuvir) tablets for oral use:US prescribing information.

[8] Feld JJ, Ramji A, Shafran SD, Willems B, Marotta P, Huchet E (2017). Ledipasvir-sofosbuvir plus ribavirin in treatment-naive patients with hepatitis C virus Genotype-3 infection:an open-label study. Clin Infect Dis.

[9] Alkaaby BA, Al-Ethawi AE (2018). The effectiveness of oral antiviral (Sofosbuvir/Ledipasvir) in treating children with HCV infection. Pak J Med Sci.

[10] Moser S, Kozbial K, Laferl H, Schutz A, Reiberger T, Schwabl P (2018). Efficacy of ledipasvir/sofosbuvir plus ribavirin for 12 weeks in patients with chronic hepatitis C Genotype-3 and compensated liver disease. Eur J Gastroenterol Hepatol.

[11] Dalgard O, Weiland O, Noraberg G, Karlsen L, Heggelund L, Färkkilâ M (2017). Sofosbuvir based treatment of chronic hepatitis C Genotype-3 infections—a Scandinavian real-life study. PloS One.

[12] Foster GR, Goldin RD, Thomas HC (1998). Chronic hepatitis C virus infection causes a significant reduction in quality of life in the absence of cirrhosis. Hepatology.

[13] Bonkovsky HL, Snow KK, Malet PF, Back-Madruga C, Fontana RJ, Sterling RK (2007). Health-related quality of life in patients with chronic hepatitis C and advanced fibrosis. J Hepatol.

[14] Ware JE, Bayliss MS, Mannocchia M, Davis GL (1999). Health-related quality of life in chronic hepatitis C:impact of disease and treatment response. Hepatology.

[15] Younossi Z, Kallman J, Kincaid J (2007). The effects of HCV infection and management on health-related quality of life. Hepatology.

[16] Wei L, Lim SG, Xie Q, Văn KN, Piratvisuth T, Huang Y (2019). Sofosbuvir-velpatasvir for treatment of chronic hepatitis C virus infection in Asia:a single-arm, open-label, phase 3 trial. Lancet Gastroenterol Hepatol.

[17] Del Rio-Valencia JC, Asensi-Diez R, Madera-Pajin R, Yunquera-Romero L, Muñoz-Castillo I (2018). Interferon-free treatments in patients with hepatitis C Genotype-3 infection in a tertiary hospital. Rev Esp Quimioter.

[18] Ramos H, Linares P, Badia E, Martin I, Gomez J, Almohalla C (2017). Interferon-free treatments in patients with hepatitis C genotype 1-4 infections in a real-world setting. World J Gastrointest Pharmacol Ther.

[19] AbouBakr O, Ezz El Regal M, Sarhan AA, El Sayed Zaki M, Noaman A (2022). Safety and Efficacy of Ledipasvir/Sofosbuvir in the Treatment of Chronic Hepatitis C Virus Infection in Treatment-Naïve Children without and with Comorbidities. Paediatr Drugs.

[20] Indolf G, Giometto S, Serranti D, Bettiol A, Bigagli E, De Masi S (2020). Systematic review with meta-analysis:the efcacy and safety of direct-acting antivirals in children and adolescents with chronic hepatitis C virus infection. Aliment Pharmacol Ther.

[21] Kawakubo M, Eguchi Y, Okada M, Iwane S, Oeda S, Otsuka T (2018). Chronic hepatitis C treatment with daclatasvir plus asunaprevir does not lead to a decreased quality of life. Inter Med.

[22] Younossi ZM, Stepanova M, Chan HL, Lee MH, Yu ML, Dan YY (2016). Patient-reported outcomes in Asian patients with chronic hepatitis C treated with ledipasvir and sofosbuvir. Medicine.

[23] Younossi Z, Stepanova M, Omata M, Mizokami M, Walters M, Hunt S (2017). Health utilities using SF-6D scores in Japanese patients with chronic hepatitis C treated with sofosbuvir-based regimens in clinical trials. Health and Quality of Life Outcomes.

[24] Fagundes RN, Ferreira LE, Pace FH (2020). Health-related quality of life and fatigue in patients with chronic hepatitis C with therapy with direct-acting antivirals agents interferon-free. PLoS One.

